# Sex hormones in COVID‐19 severity: The quest for evidence and influence mechanisms

**DOI:** 10.1111/jcmm.18490

**Published:** 2024-06-23

**Authors:** Haiqing Xiao, Jiayi Wei, Lunzhi Yuan, Jiayuan Li, Chang Zhang, Gang Liu, Xuan Liu

**Affiliations:** ^1^ State Key Laboratory of Vaccines for Infectious Diseases, Xiang An Biomedicine Laboratory & Center for Molecular Imaging and Translational Medicine, Institute of Artificial Intelligence, School of Public Health Xiamen University Xiamen China; ^2^ Clinical Center for Biotherapy, Zhongshan Hospital (Xiamen) Fudan University Xiamen China

**Keywords:** COVID‐19, gender, hormone, immune response, SARS‐CoV‐2

## Abstract

Studies have reported variable effects of sex hormones on serious diseases. Severe disease and mortality rates in COVID‐19 show marked gender differences that may be related to sex hormones. Sex hormones regulate the expression of the viral receptors ACE2 and TMPRSS2, which affect the extent of viral infection and consequently cause variable outcomes. In addition, sex hormones have complex regulatory mechanisms that affect the immune response to viruses. These hormones also affect metabolism, leading to visceral obesity and severe disease can result from complications such as thrombosis. This review presents the latest researches on the regulatory functions of hormones in viral receptors, immune responses, complications as well as their role in COVID‐19 progression. It also discusses the therapeutic possibilities of these hormones by reviewing the recent findings of clinical and assay studies.

## 1 INTRODUCTION

The coronavirus disease 2019 (COVID‐19) pandemic, triggered by the severe acute respiratory syndrome coronavirus 2 (SARS‐CoV‐2), has led to over 6 million fatalities globally (https://coronavirus.jhu.edu/map.html). Researches have highlighted significant gender‐based disparities in the mortality and critical illness rates due to COVID‐19. Early data from the China CDC revealed that men had a higher fatality rate (2.8%) in comparison to women (1.7%).^1^ This observation has been consistently validated by further population‐wide studies and meta‐analyses. Despite no evident difference in infection rates between genders according to a worldwide meta‐analysis of 3,111,714 cases, data showed that men are significantly more likely to require intensive care unit (ICU) admission (odds ratio, OR = 2.84) and face a higher death risk (OR = 1.39).^2^ Moreover, a cohort study of 18,647 patients found that male gender (adjusted odds ratio, aOR 1.896) and age (aOR 1.065) were associated with increased risks of ICU admission and all‐cause mortality.^3^ Interestingly, observational studies have also pointed out that asymptomatic pregnant women with COVID‐19 tended to develop more severe symptoms in the postpartum period, aligning with the substantial hormonal fluctuations following childbirth.^4^, ^5^ Furthermore, the association between gender and the outcomes of COVID‐19 was found to vary with age. In the prepubertal age group, there was no discernible difference in the risk of infection or mortality between girls and boys. However, in the adult premenopausal age group, females exhibited a significantly higher risk of infection than males in the same five‐year age strata. However, in peri‐ and postmenopausal females, the ratio was found to have changed once more, with infection susceptibility converging with that of males.^6^ Aging, childbirth and menopause bring about substantial alterations in sex hormones, believed to be contributing to the sex differences observed for COVID‐19 severity. In line with this hypothesis, there is a relationship among SARS‐CoV‐2 exposed men with complications of COVID‐19 with metabolic and sex hormone imbalances.^7^, ^8^ A cohort study illustrated that testosterone, estradiol (E2) and insulin‐like growth factor 1 (IGF‐1) levels, regulated by sex hormone signalling, in blood serum, correlate with disease severity in COVID‐19 patients.^9^ Additionally, a study focusing on Gender Dysphoria (GD) patients found that female‐to‐male (FtM) individuals undergoing testosterone treatment had a 3.46 times greater infection rate than male‐to‐female (MtF) individuals receiving E2 and anti‐androgens therapy.^10^ The finding provided additional evidence for the hypothesis that sex hormones contribute to susceptibility to and progression of COVID‐19. Indeed, the critical impacts of sex hormones on infectious and immune‐related diseases has been underscored by numerous studies,^11^, ^12^, ^13^ and with the advent of the COVID‐19 crisis, it has garnered increased research interest. An expanding body of evidences suggest that gonadal hormonal influences, particularly E2, progesterone and testosterone, may underlie sex differences in COVID‐19, although results from these studies have not been uniform.

**FIGURE 1:**
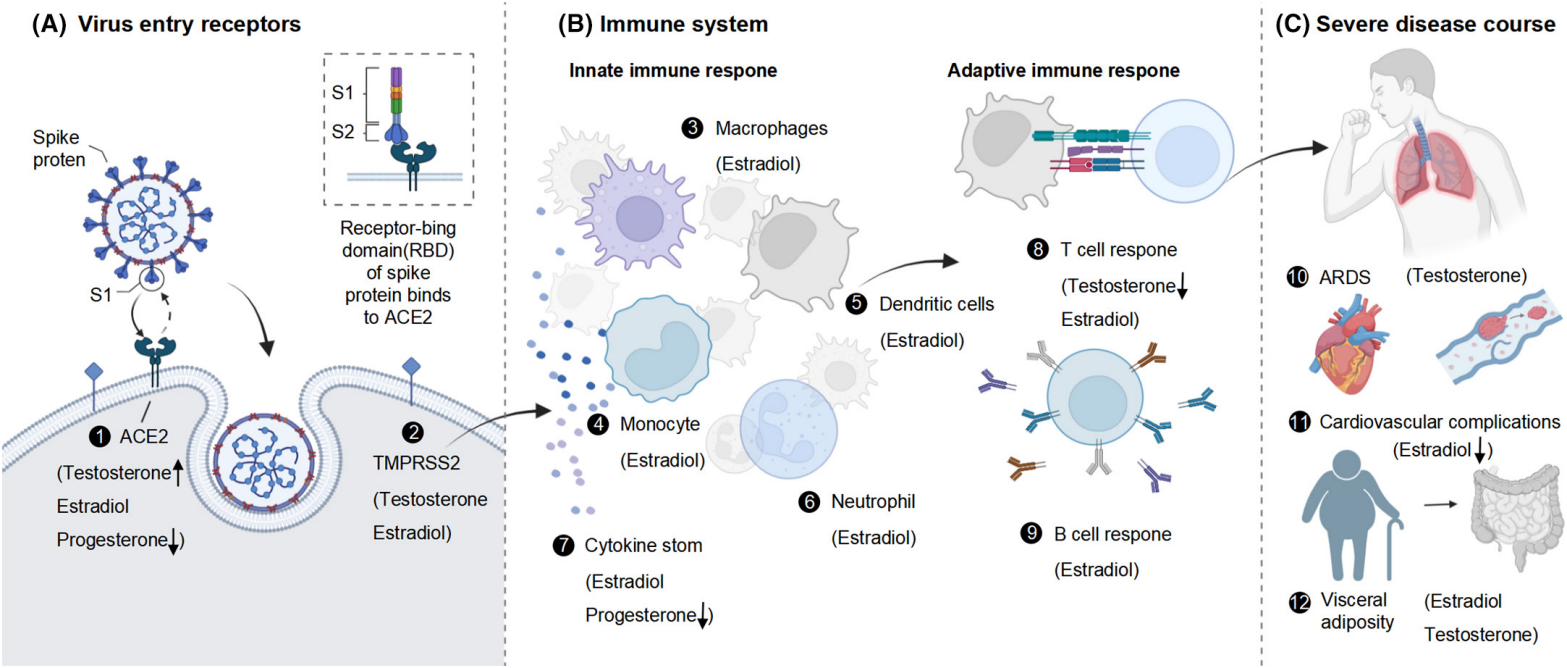
Effects of sex hormones on the course of severe COVID‐19. (A) Potential effects of sex hormones on SARS‐CoV‐2 infection by affecting the expression of viral entry receptors. Both testosterone, estradiol and progesterone impact the viral entry receptors ACE2 (1) and TMPRSS2 (2), with testosterone reported to increase ACE2 expression and progesterone reported to decrease ACE2 expression. (B) Sex hormones potentially modulate a wide range of immune cells, which may lead to differences in immune responses to viruses in hosts of different sexes. This fragment describes the innate immune response (3–7) and the adaptive immune response (8–9), both of which have been reported to be affected by estradiol. However, the direction of the effect is controversial. Additionally, testosterone has been shown to suppress the T‐cell response. (C) The impact of sex hormones on complications such as ARDS, coronary heart disease and VAT. Estradiol has been found to have an effect on the development of ARDS, but different reports have shown inconsistent conclusions regarding the direction (10). Additionally, estradiol has been reported to inhibit coronary artery disease (11). Both estradiol and testosterone have been found to influence the development of VAT, but the direction of adjustment has not been consistently reported (12). Key events affecting the prognosis of COVID‐19 are illustrated in the figure (1). The sex hormones that affect the events are listed in parentheses. Upward (downward) arrows indicate promotion (downregulation) of these events by the sex hormone. Sex hormones without arrows have been reported to have an impact on the event, but different reports draw different conclusions and are inconclusive. Angiotensin‐converting enzyme 2 (ACE2); TMPRSS2, transmembrane protease serine 2; ARDS, acute respiratory distress syndrome; VAT, visceral adiposity.

The review presents the latest researches on the regulatory functions of hormones in viral receptors, immune response and complications as well as their role in COVID‐19 progression (Figure 1). It also discusses the potential therapeutic applications of these hormones based on recent findings in the clinic.

## 2 SEX HORMONES AND COVID‐19

### 2.1 Androgens and COVID‐19 severity

Recent researches have suggested that initial levels of androgens might correlate with unfavourable outcomes in COVID‐19 patients. In a prospective cohort study comprising 66 men with severe COVID‐19 and 24 men with less severe disease, researchers quantified patients' testosterone concentrations at the initial visit to the clinic and identified that patients with severe COVID‐19 exhibited significantly lower baseline testosterone levels compared to those with milder symptoms (53 ng/dL vs. 151 ng/dL).^9^ The comparisons were adjusted for age, body mass index (BMI), race, smoking history, and comorbidities at baseline.^9^ However, another study found a significant positive association between serum total testosterone levels measured several years before exposure to SARS‐CoV‐2 and the risk of death from COVID‐19 after adjusting for confounding variables such as lifestyle, underlying diseases and other variables.^14^ Other large cohort multicentre studies have also shown that exogenous lowering of testosterone levels is beneficial in relieving severe COVID‐19 disease. In a retrospective study conducted by Montopoli et al. on the pathology of 9280 patients with SARS‐CoV‐2 infection in 68 hospitals, it was found that that patients with prostate cancer undergoing androgen suppression therapy (ADT) showed a reduction in infection rates compared to their counterparts not on ADT (OR 4.05; 95% CI 1.55 ~ 10.59).^15^ Long‐term treatment of benign prostatic hyperplasia (BPH) with 5‐alpha reductase inhibitors (5ARIs) has also been associated with a reduction in recurrence and mortality in male patients with COVID‐19.^16^ Yet, evidence from clinical trials regarding the efficacy of ADT in critical COVID‐19 cases remains scarce. A randomized clinical trial was conducted to evaluate the efficacy of a hormonal intervention for the treatment of severe COVID‐19 disease in veterans. The trial involved 96 patients at 14 hospitals, who were randomly assigned to receive either degarelix (a single subcutaneous dose of 240 mg) or a saline placebo intervention. The results demonstrated that there was no statistically significant difference between patients in the degarelix and placebo groups (30.6% vs. 26.5%; *p* = 0.67). Treatment with the anti‐androgenic agent degarelix failed to alleviate COVID‐19 severity.^17^ Likewise, a randomized phase 2 clinical trial of enzalutamide for the treatment of COVID‐19 included 42 patients hospitalized with COVID‐19 prior to safety assessment. The trial was terminated early due to the finding that enzalutamide‐treated patients required longer hospital stays (hazard ratio, HR for discharge 0.43, 95% CI 0.20 ~ 0.93).^18^ In fact, diminished serum testosterone might signal organ compromise and the overall prognosis for individuals infected with SARS‐CoV‐2, reflecting the endocrine profile of those in critical condition.^19^ Observational studies have underscored a significant reduction in testosterone levels among critically ill males in comparison to those less severely affected. In a prospective cohort study, testosterone, E2 and IGF‐1 concentrations were measured on the day of consultation and on days 3, 7, 14 and 28 after admission. Testosterone levels dropped by 64.9% upon hospital admission, with a further decline of 84.1% observed within the subsequent 3 days.^9^ In a prospective cohort study of 358 COVID‐19 patients published last year, patients underwent venous serum collection on the first day of their visit. Venous serum samples were obtained from each patient diagnosed with COVID‐19 in a tube of at least 5 mL on the first day of hospitalization. Patients requiring ICU treatment showed lower testosterone levels compared to non‐ICU patients (64 ng/dL vs. 286 ng/dL).^20^ Although some studies have shown conflicting results, it may be due to confounding factors such as race and age of the study population, as well as experimental design such as correction factors and sample size. Overall, these studies suggest that higher baseline androgen levels and lower testosterone levels in patients at disease onset are associated with organ damage and poorer prognosis. However, clinical trials of anti‐androgen therapy have not shown favourable results, which may be related to the complex mechanism of action, drug dosage, and patient population, among other factors. Therefore, more in‐depth studies are needed to investigate the mechanisms associated with the effects of testosterone and severe COVID‐19 disease.

### 2.2 E2 and COVID‐19 severity

Researches have demonstrated that E2 is associated with inflammation levels in scenarios involving acute infectious diseases, significantly influencing both innate and adaptive immune responses.^21^ In a retrospective study of 1902 female COVID‐19 patients, researchers analysed the association between menstrual status, sex hormones and immune‐inflammation‐related cytokines and disease prognosis in COVID‐19 patients.^22^ Anti‐Müllerian hormone (AMH) and E2 might be considered possible preventive agents against the severity of COVID‐19, showing a negative correlation.^22^ Multiple studies have provided evidences that supplemental oestrogen confers a protective effect on COVID‐19 in postmenopausal females, with particular emphasis on the effectiveness of E2.^6^ Electronic health records were used to analyse age‐related sex differences in a large international COVID‐19 cohort of 68,466 cases. The results showed that the risk of death was reduced by more than 50% in women under 50 years of age treated with E2 (OR 0.33, 95% CI 0.18 ~ 0.62). In contrast, in younger premenopausal women (15 ~ 49 years), E2 treatment had no effect on the risk of COVID‐19 death, probably because of higher endogenous E2 levels. A significant population‐based study further supports the hypothesis of a protective effect of E2 in COVID‐19. In women ≥50 years of age, antiestrogen therapies (AETs) decreased the occurrence of serious COVID‐19 in patients suffering from hormone‐driven cancers (HDCs) (adjPOR = 0.66;90% CI: 0.48 ~ 0.91).^23^ However, no significant associations with SARS‐CoV‐2 infection, hospitalization or death were found in all patients with HDCs receiving AETs. In a retrospective cohort study, sex hormone levels (E2 and testosterone) were recorded in male and female COVID‐19 patients (*n* = 50) admitted to the ICU and compared with non‐COVID‐19 control patients (*n* = 42) admitted to the ICU. Testosterone levels were significantly lower in critically ill male COVID‐19 patients compared to controls. However, it's worth noting that sex hormone values among severely affected women with COVID‐19 did not show statistically significant differences compared to female patients with mild disease, but a similar trend towards higher E2 levels was observed.^8^ The phenomenon can be attributed in part to the complexity of E2 and COVID‐19 interactions, reflecting the interplay among different subtypes of E2, age, and reproductive status. In these studies, E2 levels which were too low seemed to be associated with a poor prognosis for COVID‐19 in older women or men. In contrast, E2 was not significantly associated with COVID‐19 prognosis when younger women were included, which may be related to the higher baseline levels in younger women. These results therefore indicate that excessively low E2 levels appear to be associated with poor prognosis in COVID‐19.

### 2.3 Progesterone and COVID‐19 severity

Progesterone, a vital steroid hormone, is crucial for maintaining pregnancies in female and is also produced and functions in males. Progesterone exhibits various functions including reducing leukocyte activation, mitigating pro‐inflammatory mediator production, regulating T‐cell differentiation, contributing to neurodevelopment and exerting anti‐inflammatory effects. It has been recognized as essential for facilitating rapid recovery from influenza A virus infection, prompting discussions about its potential benefits in immune dysregulation during SARS‐CoV‐2 infection. Data from a clinical trial in 2021 revealed that administering progesterone subcutaneously (100 mg subcutaneously twice daily for up to 5 days) ameliorated the clinical status of 20 male patients severely impacted by SARS‐CoV‐2.^24^ Compared with controls, patients in the progesterone group had an overall improvement in mean clinical status score of 1.5 points on a 7‐point rating scale from baseline to day 7.^24^ Support for this theory is further enhanced by experimental research, which suggested that progesterone, given in a dose‐responsive manner, can counteract weight loss and mitigate severe pneumonia in hamsters infected with SARS‐CoV‐2.^25^ Furthermore, recent statistics from China on 118 cases of COVID‐19 pregnant women revealed that the gravest instances of COVID‐19 among pregnant women are more likely postpartum, a period marked by a swift decrease in progesterone levels.^26^ Analysis from the U.S. Centers for Disease Control and Prevention (CDC)'s tracking database showed, among 8207 early‐stage female pregnancies during an outbreak, adjusted for age, underlying disease and ethnicity, pregnant patients had an increased likelihood of experiencing severe disease, although the risk of death was similar to that of non‐pregnant women.^27^ However, one smaller independent study has reported that pregnancy does not have a negative impact on COVID‐19 outcomes in pregnant women exposed to SARS‐CoV‐2.^28^ The result may be related to the small sample size. Overall, the evidence from these studies suggested that excessively low progesterone may be associated with a poor prognosis for COVID‐19. Both animal studies and clinical trials suggest that progesterone supplementation in men with low progesterone levels may improve the severe symptoms of COVID‐19. However, the results were not consistent across studies. Potential explanations for this include inconsistencies in the ethnicity of the study populations, sample sizes, and grading criteria for severe disease. Therefore, the effect of progesterone on COVID‐19 severity needs to be further investigated in randomized controlled trials with larger samples.

## 3 SEX HORMONES AND EXPRESSION OF VIRAL RECEPTORS

### 3.1 ACE2 and TMPRSS2 in COVID‐19 infections

SARS‐CoV‐2, the virus responsible for COVID‐19, is an enveloped RNA virus with spike glycoproteins (S‐proteins) on the surface. To infiltrate host cells, SARS‐CoV‐2 depends on surface proteins of human respiratory tract epithelial cells, notably the transmembrane serine protease 2 (TMPRSS2) and ACE2^29^, ^30^ (Figure 1). The activation and subsequent cleavage of S‐proteins by TMPRSS2 are critical‐for the fusion of the viral envelope and the cell membrane of the host.^31^ As a type I transmembrane glycoprotein with carboxypeptidase activity, ACE2 facilitates intracellular entry. These proteins exhibit varying expression levels across tissues.^32^

During the progression of SARS‐CoV‐2 infection, elevated ACE2 levels could facilitate the entry of the virus into the body, potentially leading to organ damage. Research has indicated that eliminating TMPRSS2 from the respiratory system impacted the initial infection site and viral dissemination in the airways, consequently diminishing lung pathology in SARS‐CoV and MERS‐CoV cases.^33^ Several inhibitors of TMPRSS2 have been pinpointed as effective in blocking the ingress of SARS‐CoV‐2 in vitro.^34^ Researches suggested that TMPRSS2 and similarly functioning proteases mediate SARS‐CoV‐2 transmission through the upper respiratory tract, while the transmission might be obstructed by the administration of camostat mesylate.^35^ In the realm of clinical investigations, patients with COVID‐19 who received treatment with camostat mesylate, a serine protease inhibitor, experienced symptom relief and faster recovery of taste and smell sensation compared to other groups.^36^ However, a randomized trial found that adding a nafamostat to the standard care regimen did not reduce the treatment duration, while a minor fraction of patients at high risk of severe COVID‐19, who required oxygen therapy, showed accelerated recovery.^37^ Indeed, TMPRSS2 relies on ACE2 for its functional activity in viral fusion. A case–control study found that the TMPRSS2/ACE2 ratio was more effective than ACE2 alone in predicting COVID‐19 severity.^38^

### 3.2 Sex‐specific expression of TMPRSS2 and ACE2

Currently, there is no unified agreement on the variances in ACE2 expression levels between genders. Previous research has suggested that gene expression of ACE2 might be more prevalent in males than in females, particularly within lung epithelial cells and airway smooth muscle (ASM) cells.^39^, ^40^ However, other studies have reported similar ACE2 expression levels across various tissues in both genders.^41^, ^42^ The debate extends to TMPRSS2 expression levels in men versus women. Meta‐analysis of single‐cell data has revealed that TMPRSS2 expression is higher in AT1 cells of males than in females,^43^ though this difference is not observed in AT2 cells, which are primary targets for SARS‐CoV‐2 infection. Furthermore, subsequent research has not identified any notable differences in TMPRSS2 expression within lung tissues between the two genders.^44^, ^45^ The genes for ACE2 and angiotensin II receptor 2 are situated on the X chromosome. In females, to preserve genetic equilibrium, one X chromosome is inactivated at the blastocyst stage, but specific genes, including those coding for ACE2 and angiotensin II receptor 2, bypass this inactivation process.^46^, ^47^ Additionally, studies using rat models have demonstrated that the SRY gene family, present on the Y chromosome in males, contributes to the enhanced activation of renin‐angiotensin‐aldosterone system (RAAS) elements, thus inhibiting the activation of the ACE2 promoter.^48^

### 3.3 Regulation of TMPRSS2 and ACE2 by sex hormones

However, hormonal factors can also impact gender‐specific disparities in TMPRSS2 and ACE2 expression and activity (Table 1). Genomic data analysis showed that E2 enhances ACE2 expression in thymus cells from mice and adenocarcinoma cells from human lung epithelium.^49^ Mononuclear macrophages were collected from 10 congestive heart failure (CHF) patients before and after 1 month of treatment with Mineralocorticoid receptor blockade (MRB) (25 mg/d). MRB suppress proinflammatory genes in patients with CHF while simultaneously increasing ACE2 mRNA expression and activity (increase of 300% and 654%).^50^ Nevertheless, in experimental studies, alveoli cells were treated with estrogenic molecules or the glycosylation inhibitor clindamycin (0.2 μM) under physiological conditions for 24 h. The result indicated that E2 disrupt glycan‐glycan interactions and glycan‐protein interactions between the human ACE2 and the SARS‐CoV‐2 thereby blocking its entry into cells.^51^ Furthermore, additional experimental studies treated human bronchial epithelial (NHBE) cells with E2.^52^ E2 suppresses ACE2 expression without affecting TMPRSS2 expression.^52^ An observational study of prostate cancer patients demonstrated that prostate carcinoma containing the TMPRSS2‐ERG fusion gene may respond to E2 signalling.^53^ Treatment with 17β‐E2 on VERO E6 cells results in a decrease in the TMPRSS2 mRNA levels, consequently lowering intracellular viral load of SARS‐CoV‐2, while ACE2 mRNA was not affected in these cells.^54^ Progesterone exhibited greater efficacy in reducing ACE2 mRNA expression in human uterine tissue compared to E2 treatment.^55^

**TABLE 1:** Regulation of TMPRSS2 and ACE2 expression by sex hormones.

Viral receptor	Sex hormone‐related treatment	Primary findings	Tissue or cell type	References
ACE2	Antiandrogen enzalutamide	Decreases ACE2 expression	Heart cells and pulmonary tissue derived from human embryonic stem cells	^7^
		Reducing TMPRSS2 expression	Human lung cells	^56^
		Moderately reduces ACE2	Mouse lung	^45^
	Testosterone	Increase ACE2 expression	Mouse kidney	^49^
	Oestrogen	Increases ACE2 expression	Mouse thymus	^49^
		Reduced ACE2 glycosylation	Mouse model	^51^
		Regulates the expression of SARS‐CoV‐2 receptor ACE2 in differentiated airway epithelial cells	Female airway epithelial cells	^52^
	Mineralocorticoid receptor blocker	ACE2 activity and mRNA expression increased	Human macrophage cells	^50^
	Progesterone	Induced ACE2 mRNA expression more than oestrogen	Mouse uteri	^55^
TMPRSS2	Antagonist enzalutamide	Did not decrease pulmonary TMPRSS2	Mouse lung	^45^
		Reduced TMPRSS2 levels	Mouse airway epithelial cells	^58^
	Leuprolide or Estradiol	In males treated with leuprolide or estradiol, TMPRSS2 levels were markedly lower	Human epithelial cells	^57^
	ERbeta agonist	Expression of TMPRSS2‐ERG decreased after ERbeta agonist treatment	Human prostate cancer cells	^53^
ACE2 and TMPRSS2	Androgen	Decreased TMPRSS2 and ACE2 expression in lung epithelial cells	Mouse lung epithelial cells	40, 41
	Castration	Reduced levels of ACE2 and TMPRSS2 in lung, seminal vesicles and small intestine; ACE2 upregulated in kidney tissue, but not TMPRSS2	Mouse systemic	^56^

ADT has believed to provide protective effects by inhibiting SARS‐CoV‐2 entry into lung epithelial cells.^41^ Study has confirmed that testosterone increases ACE2 expression. Researchers investigated the regulation of ACE2 expression by oestrogen and testosterone using primary human ASM cells isolated from normal men and women as a model. Exposure to testosterone for 24 h elevated ACE2 levels in exposed human airway epithelium from both genders.^40^ A recent comprehensive screening of nearly 1500 FDA‐approved drugs aimed to identify compounds reducing ACE2 levels in normal cell cultures. Treatment with antiandrogen drugs reduced ACE2 expression in cardiomyocytes derived from stem cells and shields pulmonary organs from SARS‐CoV‐2 infection.^7^ Additionally, in an animal experiment, the researchers took lungs from female and male mice treated with a control diet or Enz for 10 days and analysed protein and mRNA expression. In mice lungs treated with the potent anti‐androgen enzalutamide, a modest decline in ACE2 gene expression was noted.^45^ Moreover, androgen deprivation through antiandrogen treatments via depot or in vitro methods leaded to decreased expression of both TMPRSS2 and ACE2 transcripts and proteins in humans.^56^ TMPRSS2 is high in the human prostate tissue and levels increase in response to androgens through targeted androgen receptor expression (r2 = 0.39; *p* < 0.001).^57^ Treatment with the anti‐androgen drug enzalutamide reduced TMPRSS2 levels in human and mouse lungs and significantly reduced SARS‐CoV‐2 entry into lung cells and infection.^58^ Nonetheless, in animal experiments, following enzalutamide treatment, TMPRSS2 expression remained unchanged in mouse lungs.^45^ In conclusion, these investigations indicate that the complex interactions between sex hormones and the expression of ACE2 and TMPRESS2 appear to vary depending on the specific organ or environment. The regulation of ACE2 and TMPRESS2 by sex hormones remains inconclusive and requires further study.

## 4 SEX HORMONES AND IMMUNE RESPONSES

### 4.1 Cytokine storm

SARS‐CoV‐2 infection triggered the development of immune responses, leading to dysregulated innate inflammation and compromised adaptive immunity in critically ill patients. This dysregulation may result in a cytokine storm, as demonstrated by the significant increase in pro‐inflammatory cytokines in patient serum, including IL‐6, IL‐1β, IL‐2, IL‐8, IL‐17, G‐CSF, GM‐CSF, IP10, MCP1, CCL3 and TNF (Figure 1). The cytokine storm may further induce respiratory failure, multiple organ failure and even shock. It is also associated with significant neutrophil and monocyte infiltrates, resulting in extensive damage to the alveoli, characterized by the formation of hyalinization and alveolar wall thickness. Ultimately, the immune‐mediated damage extended to conditions such as splenic atrophy and lymph node necrosis, which have been observed in deceased patients.^59^

### 4.2 Gender differences in immune responses

Significant sex disparities exist in both innate and adaptive immune responses. Typically, females exhibit stronger immune responses to pathogens than males. The sexual dimorphism arises from several factors. Firstly, experimental animal studies have demonstrated that females possess a greater count of most leukocyte subpopulations, such as plasmacytoid dendritic cells (pDCs).^60^, ^61^, ^62^ Females demonstrate heightened activity of cytotoxic T‐cells^63^ and elevated levels of immunoglobulins.^64^ Conversely, male COVID‐19 patients often have lower lymphocyte counts, elevated neutrophil to leukocyte ratios, and higher levels of C‐reactive plasma protein in serum than their female counterparts.^65^ It has been noted that female patients with viral infections tend to display a more robust cytokine response, while male patients infected with SARS‐CoV‐2 often show frequently have higher levels of pro‐inflammatory cytokines, such as IL‐8. This variance in cytokine response might play a role in the poorer prognosis of COVID‐19 cases in males. Additionally, T‐cell activation tends to be subdued in males, which correlates with adverse outcomes in COVID‐19 patients.

### 4.3 Sex hormones and the immune response

Discrepancies in circulating sex hormone levels may underlie gender‐related responses to infections. E2 enhances the expression of genes crucial for the activation and longevity of B cells, including cd22, shp‐1, bcl‐2, and vcam‐1, thus enhancing immune system responses.^66^, ^67^ However, in the mouse model, testosterone appeared to inhibit the recruitment of eosinophils and neutrophils and reduce the production of IgE.^68^

Additionally, sex hormones significantly influence cytokine production, leading to gender‐specific variations in immune reactions. Numerous studies have illustrated that E2 enhances toll‐like receptor 7 (TLR7)‐mediated type I IFN responses in pDCs.^69^ In a clinical trial, postmenopausal women treated with E2 exhibited an increased production of IFN‐α and TNF‐α by pDCs following TLR7 and TLR9 stimulation.^69^ Furthermore, ovariectomized mice produced less IFN‐α and TNF‐α by the pDCs after TLR7 stimulation, which correlated with E2 receptor expression in the haematopoietic compartment of mice.^69^ In addition, other studies have shown that E2's influence on pDC differentiation, the promotion of transcription factors IRF4 and IRF5, and an enhanced prevalence of pDCs generating IFN‐α and TNF‐α post‐TLR activation. The elimination of E2 receptors in mouse pDCs was found to lower Irf5 mRNA expression and reduce the proportion of pDCs secreting IFN‐α/IFN‐β.^70^, ^71^ Additionally, in vitro experiments have shown that treating human pDCs with dihydrotestosterone (DHT) diminishes IFN‐α output in reaction to TLR7 activation.^72^ Type III IFN exhibits more localized activity at mucosal sites than type I IFN, avoiding widespread inflammatory processes.^73^ Furthermore, studies have demonstrated that E2 deprivation inhibits IFN‐γ and IL‐17 production by CD4^+^ T cells while increasing systemic levels of Tregs.^74^ Increasing estradiol concentrations in vitro promoted the switch of naïve CD4^+^T cells into Th1 cells; high physiological estradiol concentrations dampening Th1 responses, promoted Tregs, and prolonged graft survival. Moreover, as a major activator of macrophages, IFN‐γ has received increasing attention from researchers. A retrospective analysis of COVID‐19 patients, examining 27 cytokines/chemokines, uncovered a direct link between IFN‐γ and E2 levels, unlike with testosterone levels. In summary, these gender‐related differences extend from innate to adaptive immunity and are influenced by the interplay of intrinsic and adaptive immune factors, often linked to sex hormone levels.

Sex hormone levels can affect the immune response, thus potentially influencing the effectiveness of the anti‐SARS‐CoV‐2 vaccine. A study investigated the immune responses to mRNA vaccination among pregnant, lactating, and non‐pregnant women. A deep sequencing analysis was conducted on humoral vaccine responses in a cohort of pregnant women, lactating women, and age‐matched non‐pregnant controls. It revealed that pregnant or lactating women required two vaccine doses to achieve immune responses comparable to those of non‐pregnant women.^75^ Furthermore, an investigation of immunological reactions following mRNA vaccination found a significantly elevated anti‐S/RBD response in female healthcare workers (HCWs) post COVID‐19 vaccination. It was noted that females experienced a pronounced and quicker reduction in this response over time when compared to males. Moreover, an intriguing positive association was discovered between the levels of plasma testosterone in male HCWs and increased anti‐S levels, hinting at a potential gender‐specific biomarker in males.^76^ These findings provided a conceptual framework for disease prevention and vaccine development targeting high‐risk populations.

### 4.4 Regulation of disease by sex hormones through immunization

Gender‐related disparities in sex hormone‐induced immune responses significantly impact COVID‐19 patient out‐comes (Table 2). A 2021 study examined plasma sex hormones (testosterone and 17β‐estradiol), sex hormone‐dependent circulating molecules (ACE2 and angiotensin 1–7), and other known biomarkers of COVID‐19 severity at admission in male and female patients with COVID‐19. The plasma biomarker levels were analysed in an aggregated and sex‐disaggregated form in relation to the severity of ARDS on admission and the occurrence of respiratory deterioration during hospitalization. The results found that upon hospital admission, male patients suffering from COVID‐19 who exhibited lower levels of plasma testosterone and lymphocytes were more likely to experience more severe ARDS and a deteriorating condition post‐admission.^77^ Several studies have been conducted to investigate the efficacy of antiandrogenic compounds, including dutasteride—a 5α‐reductase inhibitor prescribed for treating BPH and alopecia—and proxalutamide, a blocker of the androgen receptor. These studies have similarly probed immune‐related responses. The studies shown that compared to a placebo, these agents expedite viral elimination, diminish viral shedding, and reduce C‐reactive protein levels in patients with mild to moderate COVID‐19 symptoms.^78^, ^79^ The disparate outcomes observed in these studies may be attributed to variations in the immune responses elicited. A 2022 multicenter trial evaluated the efficacy and safety of raloxifene in the treatment of adult patients with mild to moderate COVID‐19. Participants were randomized in a 1:1:1 ratio to receive oral placebo, raloxifene 60 mg, or raloxifene 120 mg (self‐administered) for up to 2 weeks. The results found that raloxifene, a specific E2 receptor modulator, increased white blood cell count and accelerated viral clearance among SARS‐CoV‐2‐infected patients.^80^ Nevertheless, due to operational difficulties, the study was discontinued during the phase 2 study segment.^80^ Furthermore, testosterone and E2 may not be the standalone factor influencing adverse outcomes in patients with COVID‐19, given that sex hormones can be interconverted. A retrospective study in symptomatic men indicated that diminished total serum testosterone levels coupled with an elevated oestrogen/testosterone ratio—a marker for systemic inflammation correlated with a heightened risk of mortality while hospitalized.^81^ The study corrected for a number of major confounders, including age, BMI, hypertension and cardiovascular disease, diabetes and malignancy. Furthermore, there is evidence indicating that progesterone may affect the observed sexual differentiation in SARS‐CoV‐2 infection rates and disease progression, highlighting a possible therapeutic strategy for managing COVID‐19. The study confirmed that progesterone is capable of inhibiting excessive pro‐inflammatory cytokine production and viral replication in the lung tissues of male hamsters infected with SARS‐CoV‐2.^25^ Another study conducted in the same year further explored the concept, revealing that viral infection can induce somatic progesterone through the hypothalamic–pituitary–adrenal (HPA) axis, which, in turn, enhances intrinsic antiviral responses in cells and mice via downstream antiviral genes.^82^ Observational studies and animal experiments have indicated that sex hormones may influence the prognosis of COVID‐19. However, clinical trials have not yielded conclusive results, suggesting that the relationship between sex hormones and COVID‐19 is complex.

**TABLE 2:** Regulation of COVID‐19 by sex hormones.

Type of study	Sex hormone‐related treatment	Species and sex	Primary findings	References
Randomized controlled trial	Raloxifene	Male/Female	Increased white blood cell counts and accelerated viral clearing	^80^
Experimental research	Progesterone	Male	Inhibit proinflammatory cytokine overproduction and viral replication in lung	^25^
Experimental research	Progesterone	Mice	Triggers downstream antiviral genes, stimulating cellular as well as mouse innate antiviral response	^82^
Experimental research	Testosterone	Male/female	Angiotensin1‐7 plasma levels and neutrophil count predicted the outcome of ARDS only in females, whereas testosterone plasma levels and lymphocytes counts only in males	^77^
Experimental research	Dutasteride	Male	In males with mild COVID‐19 symptoms undergoing early therapy with nitazoxanide and azithromycin, treatment with dutasteride reduces viral shedding and inflammatory markers compared to males treated with a placebo	^78^
Investigational study	Androgen receptor antagonist, Proxalutamide	Male	Significantly accelerated viral clearance on Day 7 in mild to moderate COVID‐19 patients versus placebo	^79^
Exploratory retrospective study	Estradiol to testosterone ratio	Male	Low total testosterone levels and elevated E2/T ratio values at admission are associated with hyperinflammatory state in hospitalized men with COVID‐19	^81^

## 5 COMPLICATIONS OF COVID‐19

### 5.1 Lung tissue damage

Severe illness and death due to acute viral pneumonia progressing to ARDS are common in patients with COVID‐19.^83^ These stages involve exudation and proliferation, which are characterized by hyaline membrane changes and microvascular thrombosis, ultimately leading to diffuse alveolar injury^84^ (Figure 1). Providing effective treatment during these stages is crucial to improving patient prognosis and preventing mortality. Studies on animals have demonstrated that male mice that have been castrated exhibit a reduction in LPS‐induced lung injury, while female mice do not.^85^ Therefore, androgens may exacerbate symptoms of lung injury and affect the prognosis of COVID‐19.

### 5.2 Cardiovascular system

During the COVID‐19 pandemic, patients may exhibit a range of extrapulmonary symptoms, including cardiovascular issues such as myocardial dysfunction, acute coronary syndromes, and thrombosis. These issues are strongly linked to mortality and result in direct damage to cardiomyocytes, virus‐mediated endothelial injury, systemic inflammation, and hypoxia.^86^ E2 is known for its protective role in the vascular system and might explain the observed sex‐based disparities in COVID‐19 fatality rates. Research on animals has demonstrated that 17β‐estradiol offers a protective shield against myocardial ischemia, notably diminishing the size of myocardial infarction.^87^ E2 signals directly through the membrane and induces vasodilation by releasing nitric oxide. Furthermore, signalling of E2 receptors plays a crucial role in maintaining the structural and functional integrity of endothelial cells by blocking the pathways leading to apoptosis.^88^ These findings highlighted the cardioprotective function of E2, reducing the susceptibility to COVID‐19‐related cardiac injury, endothelial inflammation, and subsequent cardiovascular complications.

### 5.3 Visceral adiposity

Research consistently demonstrates a heightened potential for serious illness and increased mortality among persons with an elevated BMI.^89^ For each 1‐mm increment of subcutaneous adipose tissue (SAT) thickness in humans, there is a 16% increase in the chance of serious disease.^90^ Adipose tissue expresses ACE2 receptors, and in obese patients with larger fat stores, these receptors are more abundant, intensifying the systemic response to SARS‐CoV‐2.^91^ Importantly, visceral obesity contributes to elevated levels of prothrombotic circulating factors, increasing susceptibility to thrombosis.^92^ Fat distribution is also influenced by sex hormones. Knocking down E2 receptors and reducing E2 signalling leads to obesity in both male and female mice.^93^ A randomized controlled trial has suggested a link between treatments involving anabolic steroids with androgenic properties and an increased accumulation of visceral fat.^94^ Consequently, SARS‐CoV‐2 infection may worsen inflammation in visceral adipose tissue (VAT).

Furthermore, the mesenteric VAT, encasing the small intestine, acts as a primary barrier against pathogen migration from the intestines to the circulatory system.^95^ Remarkably, more than half of COVID‐19 patients with positive faecal SARS‐CoV‐2 test results have reported gastrointestinal discomfort, indicating the virus's impact on intestinal cells.^96^ Furthermore, single‐cell RNA sequencing analyses from samples of healthy individuals have revealed elevated expressions of ACE2 and TMPRSS2 in the intestinal epithelium following infection by SARS‐CoV‐2.^97^ These findings suggested that viruses may disrupt the intestinal barrier, triggering an immune‐inflammatory response and exacerbating local inflammation. In conclusion, the outcomes of COVID‐19 are influenced to varying degrees by the complex interactions between sex hormones, immune responses, and immune metabolism.

## 6 CONCLUSIONS

In conclusion, differences of sex hormones play a role in the observed disparities of SARS‐CoV‐2 outcomes between genders. However, consensus is lacking in the existing evidence, and the intricate relationship between viruses and sex hormones make it challenging to clearly define how sexual steroids influence the progression of COVID‐19. Present clinical data on the impact of sexual hormones on the mitigation of COVID‐19 do not support for the use of E2 or discontinuation of these agents in COVID‐19 patients. Therefore, further prospective trials are required to fully grasp the enduring influence of sex hormones on the progression of COVID‐19. Several relevant trials have delved into how sex hormones affect multiple facets of COVID‐19, including their role in the regulation of ACE2 and TMPRSS2, immune reactions, and the risk of developing complications such as heart disease. However, these reports yield inconsistent findings, and a thorough exploration of the underlying mechanisms and hypotheses is required, utilizing advanced analytical methods such as multi‐omics. In addition, research focusing on the impact of sex hormones across different strains of SARS‐CoV‐2 is scant. Lastly, the influence of sociocultural gender on an individual's life experiences, immunization awareness, and medical conditions indirectly affects COVID‐19 outcomes. Therefore, studying both biological and socio‐cultural gender differences is crucial to enhance our understanding and management of the pandemic.

The outcomes of these animal experiments or observational studies support the prospect of clinical translation of sex hormones. In addition, several studies reviewed in this article demonstrate the impact of sex and age stratification on outcomes. These results serve as a reminder of the importance of stratifying populations for age, sex, etc., in the design of clinical trials. However, there is currently no evidence to support the use of oestradiol or the discontinuation of androgens in patients with COVID‐19 in the reports of clinical studies of the effect of sex hormones on COVID‐19 regression. The expected effects of clinical trials include a significant reduction in the risk of patient death after sex hormone intervention in critically ill patients with COVID‐19 compared with the placebo group. Nevertheless, there may be potential risks, including the possibility that the intervention may have the opposite effect or that the sex hormone intervention group may experience adverse effects. Several current research clinical trials have been terminated early due to failure to achieve the expected outcomes or the development of side effects, which is in line with ethical considerations. However, the dosage of sex hormone therapy may also affect the outcome of the disease due to species differences as well as individual differences, which may also be a significant challenge in the clinical translation of sex hormones. Due to ethical constraints, it was not possible to explore dosage in humans. Consequently, there is a need to develop relevant algorithms to translate doses from animals to humans, which could significantly advance the clinical translation of sex hormones.

## AUTHOR CONTRIBUTIONS

**Haiqing Xiao:** Investigation (lead); visualization (lead); writing – original draft (lead). **Jiayi Wei:** Investigation (lead); visualization (equal); writing – original draft (lead). **Lunzhi Yuan:** Conceptualization (supporting); visualization (supporting). **Jiayuan Li:** Investigation (supporting); writing – original draft (supporting). **Chang Zhang:** Investigation (supporting); visualization (supporting); writing – review and editing (supporting). **Gang Liu:** Conceptualization (equal); investigation (lead); project administration (lead); writing – review and editing (equal). **Xuan Liu:** Conceptualization (lead); funding acquisition (lead); project administration (lead); writing – review and editing (equal).

## FUNDING INFORMATION

This research was funded by the National Natural Science Foundation of China (32201152, 81925019, U1705281, U22A20333), the Major State Basic Research Development Program of China (2017YFA0205201), the Fundamental Research Funds for the Central Universities (20720190088, 20720200019), the Program for New Century Excellent Talents in University, China (NCET‐13‐0502), Shenzhen Natural Science Foundation (JCYJ20220530143407016). The funders had no role in the study design, data collection and analysis, decision to publish, or preparation of the manuscript.

## CONFLICT OF INTEREST STATEMENT

The authors declare no conflict of interest.

## Data Availability

All data is referred to in the references.
